# Conductive cross-section preparation of non-conductive painting micro-samples for SEM analysis

**DOI:** 10.1038/s41598-022-21882-1

**Published:** 2022-11-16

**Authors:** Victory Armida Janine Jaques, Eva Zikmundová, Jiří Holas, Tomáš Zikmund, Jozef Kaiser, Katarína Holcová

**Affiliations:** 1 Institute of Geology and Palaeontology, Faculty of Science, Charles University, Albertov 6, 12843 Praha 2, Czech Republic; 2 CEITEC - Central European Institute of Technology, Brno University of Technology, Purkyňova 656/123, 612 00 Brno, Czech Republic

**Keywords:** Characterization and analytical techniques, Imaging techniques, Scanning electron microscopy, Solid Earth sciences

## Abstract

Scanning electron microscopy (SEM) is a common method for the analysis of painting micro-samples. The high resolution of this technique offers precise surface analysis and can be coupled with an energy-dispersive spectrometer for the acquisition of the elemental composition. For light microscopy and SEM analysis, the painting micro-samples are commonly prepared as cross-sections, where the micro-sample positioned on the side is embedded in a resin. Therefore, the sequence of its layers is exposed after the cross-section is polished. In common cases outside of cultural heritage, a conductive layer is applied on the polished side, but in this field, the measurements are mostly done in low-vacuum SEM (LV-SEM). Although the charging effect is reduced in LV-SEM, it can still occur, and can hardly be prevented even with carbon tape or paint. This work presents two conductive cross-section preparation methods for non-conductive samples, which reduce charging effects without impairing the sample integrity.

## Introduction

Scanning electron microscopy (SEM) is a widely used analytical technique in several fields, such as engineering^1^, forensics^1–3^, geology^4^, biology^5,6^ and medicine^7,8^. In the field of cultural heritage, the SEM analysis is an essential part of the examination of painting micro-sample cross-sections^9–14^. However, the analysis of such samples is complex due to their non-conductivity, uniqueness, and both structural and material heterogeneity.

A SEM measurement is carried out in a vacuum chamber, where a convergent electron beam, created by electromagnetic lenses to focus and remove aberrations of the image, hits the surface of the sample^15^. The interaction of the beam with the sample surface produces several signals. The outputs of these signals can be interpreted with different detectors and give various information, such as the chemical composition (Energy-Dispersive Spectroscopy—EDS), which is mostly used in the study of painting micro-samples, the present phases (Back-Scattered Electrons—BSE), the texture/topography (Secondary Electrons—SE), the crystalline structure and the orientation of particles. The detailed principles are discussed elsewhere^15,16^.

**Figure 1:**
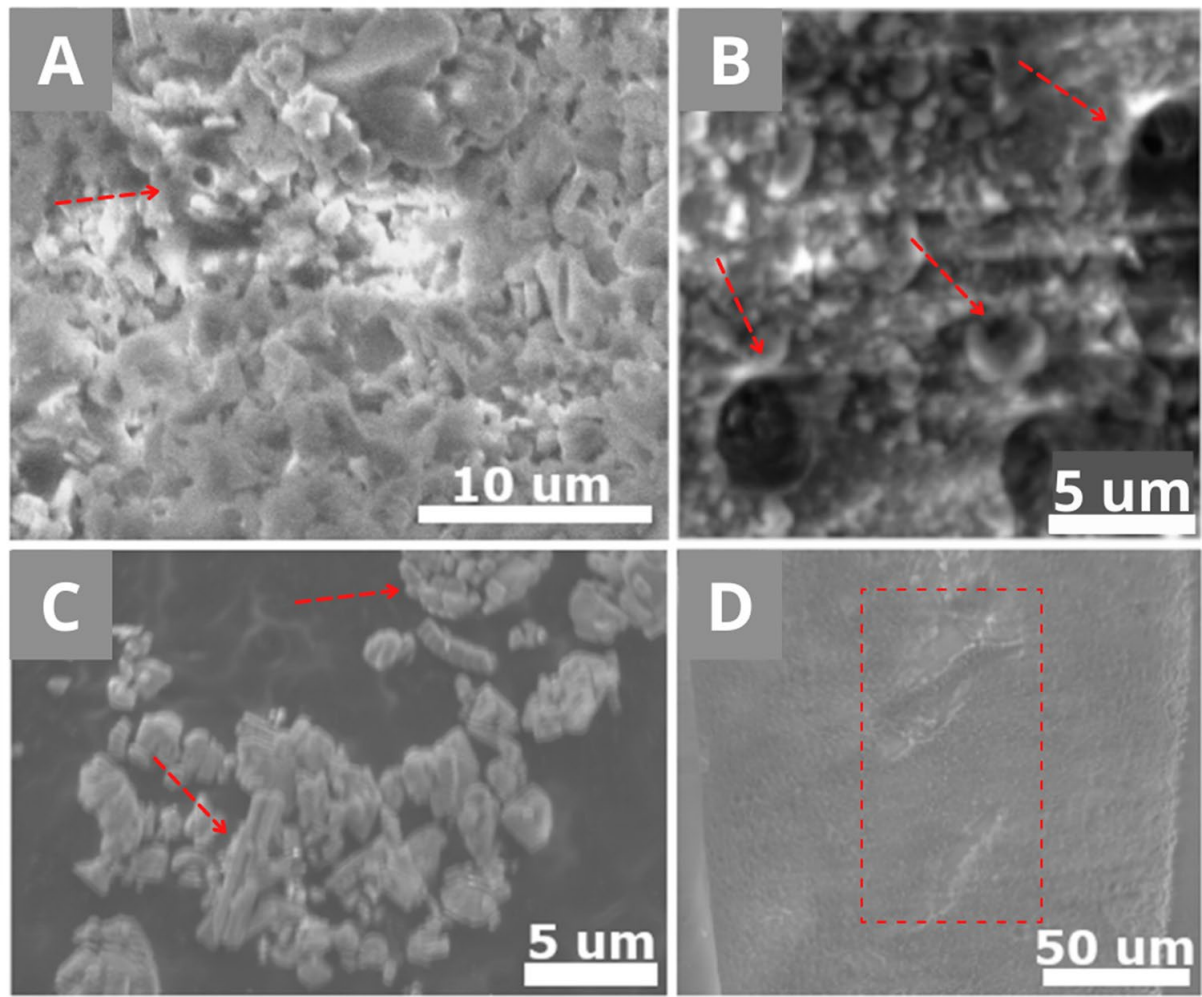
MIRA3 XMU SEM-SE images of charging effects. (**A**) and (**C**) are chalk powder composed of calcareous nannofossils; (**B**) and (**D**) show the surface of a foraminifera Uvigerina sp. The red arrows and frame show the material planned to be studied. **(A)** Uneven brightness creates bright spots and too dark areas, hiding features of the sample. **(B)** Bright lines created by charging do not allow the interpretation of the homogeneity of the surface. **(C)** Image distortion modifies the visualisation of the sample and renders its interpretation impossible. **(D)** Lack of stereoscopic sense hinders the topographical analysis and shape observation.

For the measurement, a voltage is applied for accelerating the electrons^15^.When the accelerated electrons hit gas molecules still present in the vacuum chamber, it shortens their mean free path and influences the image quality negatively^15^. High-pressure environments have fewer gas molecules and are preferred, but a charging effect may occur on non-conductive samples when the charge of the electrons is not balanced^17^. The charging effect is a concentration of electrons at a specific place, creating uneven brightness (Fig. 1A), bright lines (Fig. 1B), image distortions (Fig. 1C) and/or a lack of a stereoscopic sense (Fig. 1D). These effects could indirectly influence the measurement of the chemical composition (bad/unclear positioning).

The preparation of samples is varied^18^, but the most common is to use directly the sample as it is^19–21^ or to prepare a thin/thick section^18^. To avoid the charging effect, both methods require either coating the non-conductive samples or using a low-vacuum mode^8^. A coating is an ultra-thin conductive layer of metallic molecules (Au, Pt) or carbon applied on the surface of the sample or its cross-section^22–24^. It equalises the charge on the whole surface, where the excess of electrons flows away from the sample^1,25–27^. The sample coating is the most common way to avoid charging in a high-vacuum mode^28^. In the field of cultural heritage, the coating is often undesired because of the multi-technical approach^9–12,14^. The coating can influence surface characteristics, e. g. impair the colours of the sample^29^ and/or the elemental composition of the sample, which are important issues regarding painting micro-samples. Moreover, the removal of such layers is done mechanically or using chemicals which can strongly interact and destroy the surface of the sample^26,27,29–34^ for further studies.

In technical fields, using a conductive resin, such as an epoxy or acrylic resin mixed with an additive conductive material, is common^35^. But these resins are mostly coloured, which impairs the light microscopical visualisation of the cross-section prior to the SEM analysis^36^. The use of conductive paint and/or tape can be helpful in some cases^37^, but cannot replace a proper coating or the use of a conductive resin, and does not work on large non-conductive samples.

The analysis of non-conductive samples which cannot be coated is mainly done in a low-vacuum environment (LV-SEM)^15,38^ or an environmental SEM (ESEM)^39^. The low vacuum reduces the charging effect, but also lowers the image resolution. In this case, the gas molecules still present in the vacuum chamber, due to the low vacuum, shorten the free mean path of the electron by producing ions that neutralise the excess of electrons and reduce the charging^15^. It should be noted that the components of a sample can have various interactions with the electrons, such as outgas and evaporation, and particularly organic matter and wet matter (coal, organic material, swelling clays)^40^. An increasing chamber contamination of these materials can affect the vacuum, thus impacting the charging effect^41^.

The cross-section preparation is common for the morphological and chemical analysis of micro-samples from paintings^42–47^. The information gained from a painting cross-section can be about the painting technique, the layering, the pigments, the grains distribution, the porosity and cracks, the colour, and the binders^47–49^, which is important for conservation, the appropriate restoration and the art comprehension^13,50–54^.

A cross-section is the embedding of a sample in a material, such as a resin^54^. The choice of the resin, the encapsulation type, and the polishing can have a serious impact on the sample and measurement. They should be, therefore, chosen carefully^55,56^.

The resin should be chosen according to its characteristics^57,58^, the planned analyses and the sample type. Among the characteristics are the curing temperature^59^ and time, transparency^60^, fluorescence^61^, shrinkage^59,62,63^, hardness, viscosity^58,64^ and additives^60,61,65–67^. Some authors have used bioplastic in the last years, but there are not enough studies on their ageing available to assess the possibility of a long-term use^68^. Khandekar^69^, Derrick et al.^64^ and Waentig^70^ addressed most of the issues about the choice of the resin and the preparation material according to the sample type. A specific requirement of painting micro-samples is to use a clear and low temperature curing resin, which discards the coloured conductive resin. A low temperature curing resin prevents the evaporation or molecular modifications of the organic components often present in painting samples, but also thermal chemical reactions between some components, thus, morphological changes. There are different types of resin encapsulations^62,63,65,66^, as well as sample fixation methods, which are discussed elsewhere^67^.

In this work, the aim was to develop a resin cross-section preparation for non-conductive samples to reduce the charging effect without impairing the sample characteristics (colour, morphology, chemical composition) with any coating or coloured resin. The suggested preparation procedures are a combination of established methods of painting micro-sample preparation, and the procedures aim to be time, cost and results effective.

## Materials and methods

### Samples

The chosen samples (Table 1) represent a fully non-conductive material (MS1, MS2) and a partially conductive one (MS3, RS1, RS2) (Table 1). MS1 and MS2 are model samples made of pure chalk and an organic binder. MS3 is a partially conductive model sample. A restorer prepared the model samples to improve the comparison with historical painting micro-samples. MS3 contains a layer of yellow earth mixed with an egg yolk (tempera technique) with iron particles which make it partially conductive on top of chalk, and a rabbit skin glue non-conductive layer.

**Table 1:** Different samples regarding their porosity and conductivity were used for the experiment. MS1 and MS2 are not conductive, while MS3, RS1 and RS2 have some metallic particles (iron) in their pigmented layers.

Sample	Pigment	Preparatory layer	Binder	Number of Layers
MS1	–	Chalk	RSG	1
MS2	–	Chalk	LO	1
MS3	Yellow earth	Chalk	RSG + T	2
RS1	Green earth	Chalk	Oil U + T	3
RS2	Yellow and green earth	Mortar	Carbonaceous	4

MS: Model sample; RS: Real sample; RSG: Rabbit skin glue; LO: Linseed oil; T: Tempera; U: Unknown.

RS1 is a real historical sample with a top green earth tempera layer and a chalk preparatory layer with an unknown oil binder. RS2 is also a real sample of a wall painting, with green and yellow earth layers on top of two carbonaceous preparatory layers. All samples are smaller than 1 mm in all directions.

### Epoxy resins

The materials used for this study are relatively easy to find and established for a cross-section preparation. Various types of epoxy resins were used for the preparation^55,71^. EpoFix (Struers, DK) was used for the direct embedding of all samples and the reference non-conductive preparation. It is a transparent dual-component cold mounting resin combining an epoxy resin with a hardener. Its curing time is approximately 12 h. It is a relatively common type of resin already used for painting micro-samples embedding^64^.

PolyFast and LevoFast (Struers, DK) are hot mounting powder resins. PolyFast is a conductive resin used for the Preparation A, and LevoFast is a particularly hard resin used for the polishing holder designed for this work.

The Reference and Preparation A were prepared in a round plastic mould (Struers, DK), which is made for a vacuum impregnation chamber (CitoVac, Struers, DK) to help remove bubbles to increase the stability and hardness of the resin. To fix the positioning of the sample, plastic clips were used.

For the other preparations, two silicone ice cube trays of 1 cm × 1 cm × 1 cm and 1 cm × 1.5 cm × 1.5 cm were tested. The two trays had different silicone textures. The small one had a porous texture, giving a final milky appearance of the resin, while the large tray gave a transparent final aspect. The small cubes better fitted the size of our micro-samples and made the positioning easier. The larger cubes were easier to handle and could be useful for large or long samples. Even though the transparency of the cubes from the larger tray made it easier to observe the samples in the resin, the smaller silicon tray was harder and, thus, easier to handle, and the milky appearance was not hindering the polishing process. In our case, the smaller ice cube tray of 1 cm × 1 cm × 1 cm for the conductive preparation was more appropriate.

### Instrumentation

The CitoPress-10 hot mounting press (Struers, DK) was used for the PolyFast and LevoFast resins.

A Leica EM ACE 600 high-vacuum coater was used for the application of a carbon coating of 30 nm in Preparation B.

The grinding and polishing of the cross-sections were done as described in Jaques and Zikmundová (unpublished). Before the sample was reached, the polishing mode was changed from wet to dry^47^.

The first observations of the cross-sections were made under the Stemi 2000-C and Stemi 508 stereomicroscopes (Zeiss, DE) and a Reichert microscope (Reichert Technologies, US) coupled to an Axiocam ERc 5 s (Zeiss, DE).

The scanning electron microscope used for this study is a MIRA3 XMU (Tescan, CZ). Both the high-vacuum (5 mbar to 9 × 10–5 mbar) mode and the low-vacuum (0.07 mbar to 5 mbar) mode with a secondary electron detector (LVSTD mode) were used.

### Cross-section preparation

The non-conductive cross-sections were prepared with the 5 samples (Table 1) as references (Table 2). Then two conductive preparations (A and B) were developed (Table 2).

**Table 2:** Overview of the preparation methods.

	Reference	Conductive A	Conductive B
Embedding type^5^	Capsule + flat	Slotted-capsuled block	Flat
Resin	EpoFix	PolyFast + EpoFix	EpoFix
Mould [cm]	2 × 1 ø | 1.5 × 1.5 × 1.5	2 × 1 ø	1 × 1 × 1
Conductive material	–	PolyFast + Cu-tape	30 nm Carbon-coating
Preparation time [h]	12	7	12

### Reference preparations

**Figure 2:**
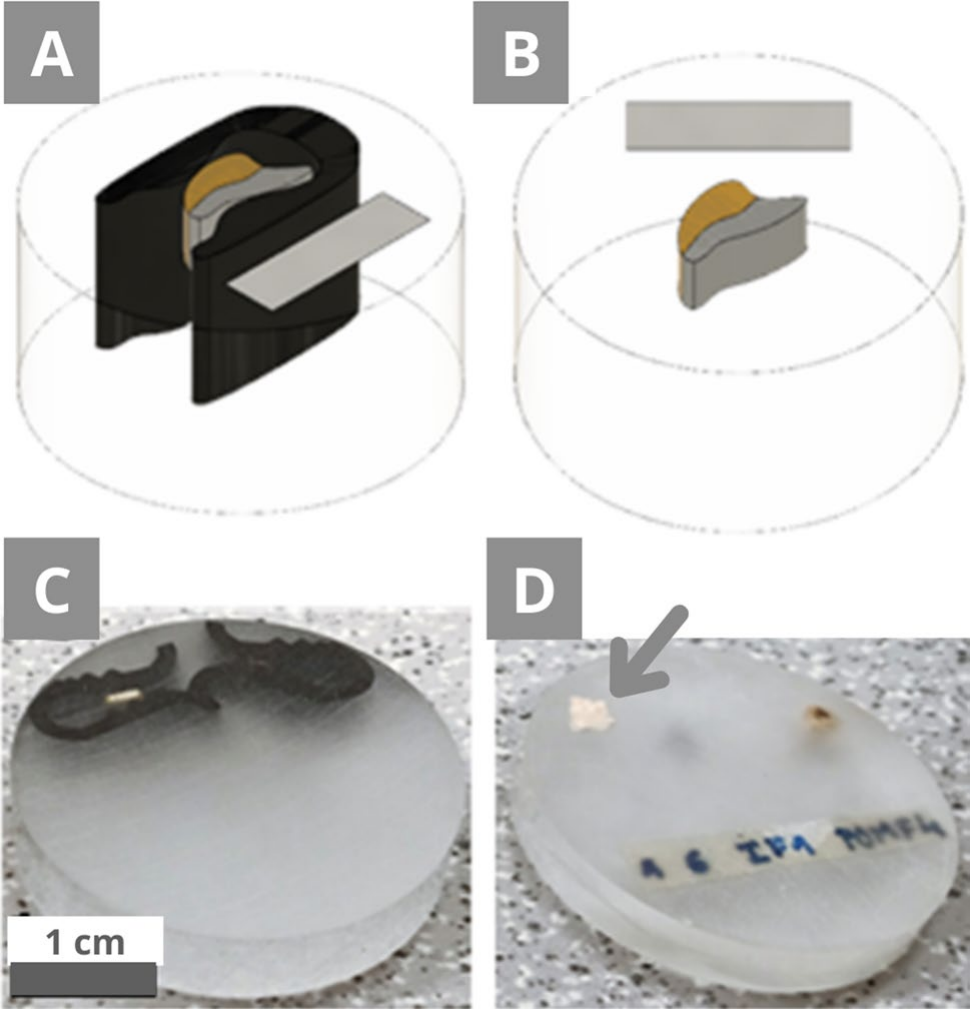
Reference preparation with the fixing clips as **(A)** a scheme and **(C)** in reality, and without the clips **(B)** as a scheme and **(D)** in reality. The grey arrow shows a part of a sample that floated to the surface of the resin (for detail see Fig. 6). The cross-section diameter is 3 cm.

The reference preparations (Fig. 2) are non-conductive, except for the specific conductive particles of the sample (MS3, RS1, RS2). The capsule reference preparation was prepared by positioning the samples and then adding the resin through the CitoVac device. The CitoVac is used to pour the resin via a plastic tube onto the micro-sample placed in a circular mould in a vacuum chamber. This technique offers cured resin with less to no bubbles and better penetration of the resin in the sample. The capsule embedding had a curing time of approximately 12 h regarding the dual-component epoxy. 12.5 g of the EpoFix per 1.5 g of the hardener were used and gently poured on top of the sample. The samples were fixed using plastic clips, which, however, moved, and had to be repositioned. A second reference preparation was made using a flat embedding with the same samples. The mould was 1.5 cm × 1.5 cm × 1.5 cm to ease manual polishing without the polishing holder. Depending on the size of the sample, it can also be useful to have a larger mould size. The sample was positioned on the surface of the already dried resin filling half of the mould. A drop of the EpoFix was applied on top of the sample and left for approximately 30 min. This lightly fixed the sample and avoided drifting or floating. Then the rest of the resin was gently poured on top. The CitoVac was not used in this case. The flat embedding gave better results for the orientation, even though its preparation is slightly more demanding. After the curing time, the embedded samples were polished with the Tegramin in an automatic mode.

### Conductive preparation A

**Figure 3:**
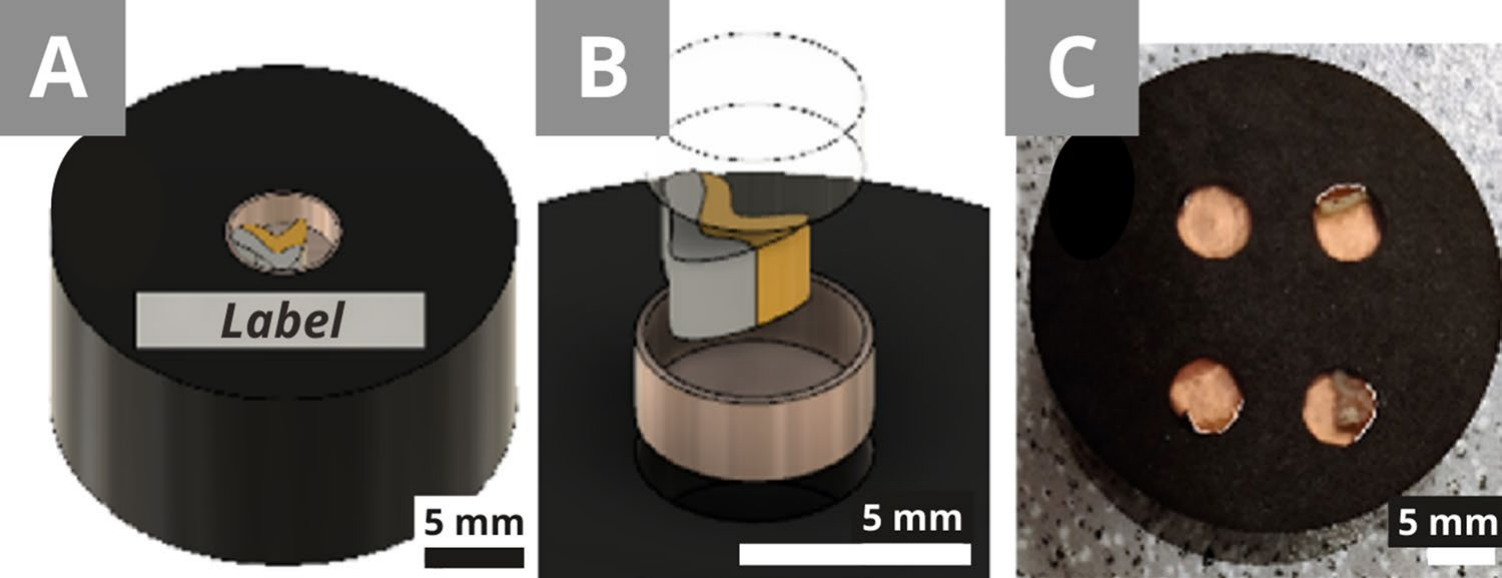
**(A)** Design of the conductive preparation A (3 cm in diameter). **(B)** A scheme of the different parts: the black PolyFast, the copper tape (bronze), the layered sample (orange/grey) and finally the transparent EpoFix. **(C)** The real preparation of a PolyFast conductive block with each of the 4 samples in a different hole with a copper tape.

Slotted-capsule embedding was chosen because the two-step preparation (hot resin then cold resin) created with small holes helps the sample positioning and partially avoids floating and drifting. The preparation using the CitoPress was done according to its instructions. This step took approximately 10 min. Four flat bottom holes of 5 mm in diameter and 2 mm deep were drilled in the PolyFast block (Fig. 3C). If the samples have a similar height (perpendicular to the bottom of the hole), more can be put in the same block, but it can make it more difficult to get an interesting surface for all of them while polishing. Therefore, a maximum of two samples of the same height in one resin block is recommended. A batch of blocks can be prepared in advance, either with or without the holes. The depth of the holes can also vary according to the size of the samples. The copper tape was added to the bottom and around the hole. One sample per hole was fixed and put into tight contact with the copper tape (Fig. 3B). The hole was finally filled with the EpoFix (Fig. 3B), and the curing was shorter due to the small amount of the resin. The cross-sections were then polished with the Tegramin.

### Conductive preparation B

**Figure 4:**
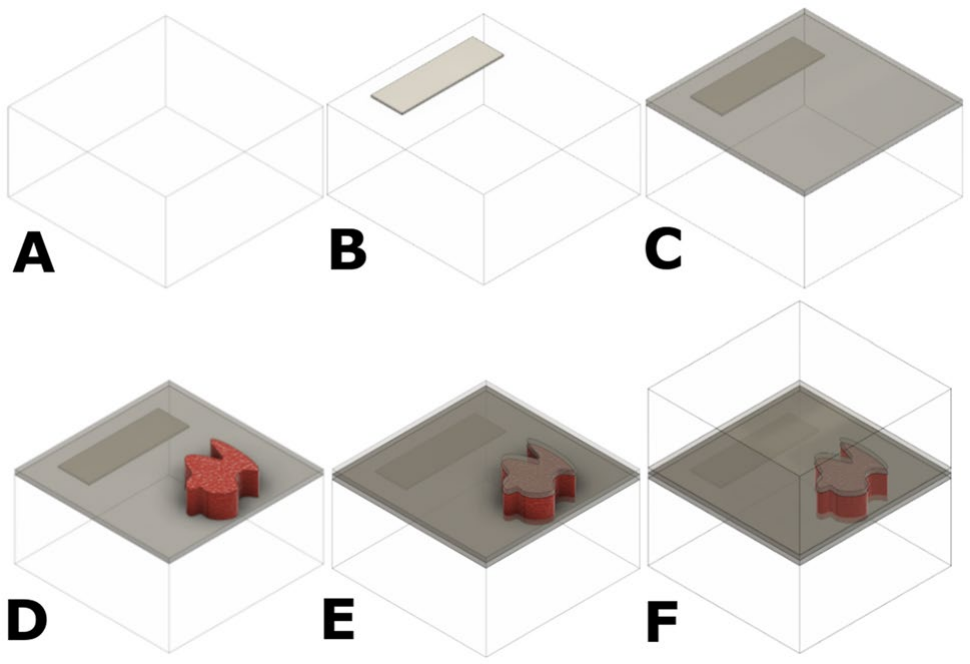
Preparation B scheme. **(A)** Cured EpoFix in 1/2 of the mould. **(B)** Label glued downwards. The label can also be added upwards after step (E). **(C)** The lower part of the resin is coated with carbon (grey). **(D)** The sample (red) is oriented on the resin ½ cube. **(E)** The surface of the sample (red) is coated with carbon (grey), which will connect with the first layer from step (C). **(F)** The rest of the mould is filled with the EpoFix and left for curing.

For the flat-embedding preparation, half of the silicone mould of 1 cm × 1 cm × 1 cm was filled with the EpoFix and left to cure similarly to the flat reference preparation (Fig. 4A). The surface of the lower part of the resin was coated with a 30 nm carbon layer with a Leica coating device (Fig. 4C). The sample was then put on top with a tiny drop of resin or a quick-dry gel glue (Fig. 4D). A high viscosity material, such as the gel glue, prevents its spreading on the resin surface and in/on the sample. Only a small part of the bottom of the sample should be glued. The glue should not interfere between the coating layer and the sample. After a short curing time of the glue, a second carbon coating is done on top of the preparation (Fig. 4E). Finally, the second layer of the resin is added to fill the mould and fix the sample (Fig. 4F). A holder specifically designed for this experiment was used for polishing the cross-sections. The first polishing was wet (using water) and then just before getting to the sample, the mode was switched to a dry mode to prevent the disaggregation of the sample components. The pressure and speed of the rotation plate were also reduced to avoid deep scratches on the surface of the resin and its melting.

### Polishing holder

**Figure 5:**
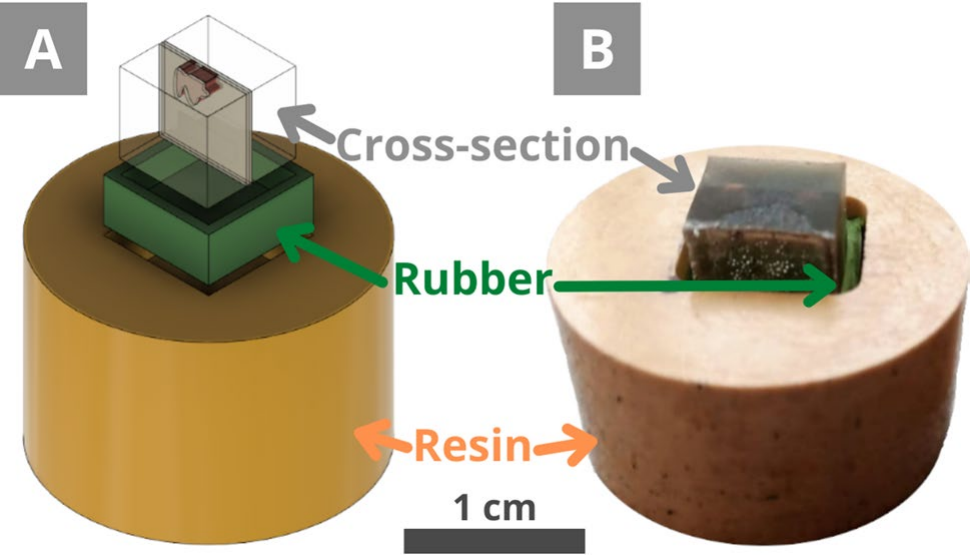
**(A)** Scheme of the holder with the LevoFast resin, a silicone rubber, and a cross-section (Fig. 4F) to be polished. **(B)** The achieved holder for the 1 cm × 1 cm × 1 cm cross-sections.

Before the analysis, cross-sections must be polished to get a clear, smooth surface of the sample without the resin on top of it. Although round moulds are typical for cross-section preparation, they are not always of the best size or shape for the sample. The smaller cubes were chosen, which are common in cultural heritage sample preparation, with a manual polishing. The Tegramin automatic mode cannot be used for such cubes because of their shape and size that does not fit the device. The cubes are also rather small, which makes it more difficult to get a flat, smooth polished surface and avoid deep scratches. Therefore, it was decided to use the Tegramin for a certain standardisation of the preparation and to avoid user errors, such as uneven surfaces and deep scratches. A polishing holder was designed to fit the cubes into the Tegramin (Fig. 5A).

The holder was made of the LevoFast resin, composed of melamine, minerals, and glass fillers. It is a harder resin than EpoFix used for embedding and, thus, more resistant to polishing. This resin is hot mounting and was prepared using the CitoPress.

A square of 1 cm × 1 cm × 0.75 cm was drilled in the centre of the round piece, and a square silicone rubber with walls of a 2 mm thickness was introduced into it (Fig. 5). The silicone rubber fixes the cross-section, allowing only small movements of it. Also, the resin can shrink during curing, preventing the final shape to be a perfect cube, hence the silicon rubber.

## Results and discussion

The preparation procedure of each method was evaluated according to the total time of the preparation, the ease of the preparation, and the visual assessment of the surface under the light and electron microscopes. The conductivity of the cross-sections prepared according to the Preparation A or B (Table 2) was evaluated in a visual comparison with the reference preparation cross-sections of a similar sample. The image quality based on the amount of charging and strength of the artefacts was evaluated during the imaging process.

### Cross-section preparation

**Figure 6:**
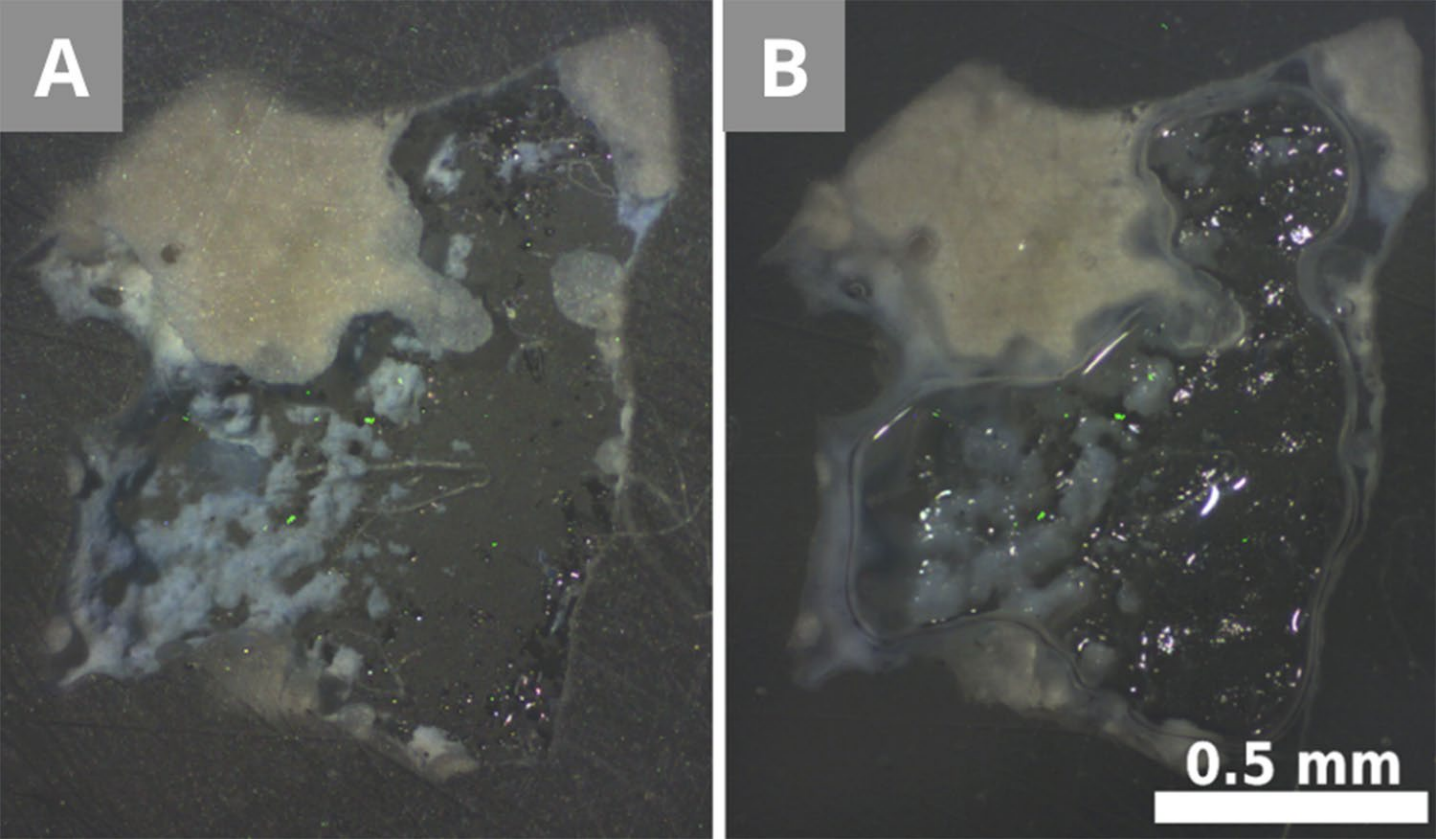
Unfixed sample MS1 that floated during the curing of the resin, and the parts of it removed during the polishing. **(A)** The scratches on the resin and the sample surface are visible. **(B)** Most scratches disappear thanks to the application of a drop of water and a coverslip over the sample, which creates an even surface.

Preparation A (Fig. 3) needs the hot resin to be prepared in advance (appr. 8 min) and the holes to be drilled in the resin (appr. 5 min), but the curing time of the transparent EpoFix is reduced from 12 h to appr. 6 h thanks to the small amount needed. The hole helps positioning the sample with copper tape and a low amount of resin, which strongly reduces the floating and drifting of the sample compared to the reference preparation. The capsule embedding (Fig. 2) offers a one-step preparation without previous planning, but the amount of the curing resin increases the risk of sample misplacement even with plastic clips. These clips are useful for flat and rather large samples, but they reduce sample visibility and cannot be used for brittle, non-flat samples. Highly porous samples floated to the surface despite multiple repositioning during the hardening of the resin (12 h). Without the clips or any other fixing, the samples floated (Fig. 2D—grey arrow + Fig. 6). One of the reference samples drifted into a horizontal position (Fig. 6), instead of staying on its side to show the layering. In our case, the sample only contained a chalk layer, but with any other sample, most stratigraphic information would have been lost.

**Figure 7:**
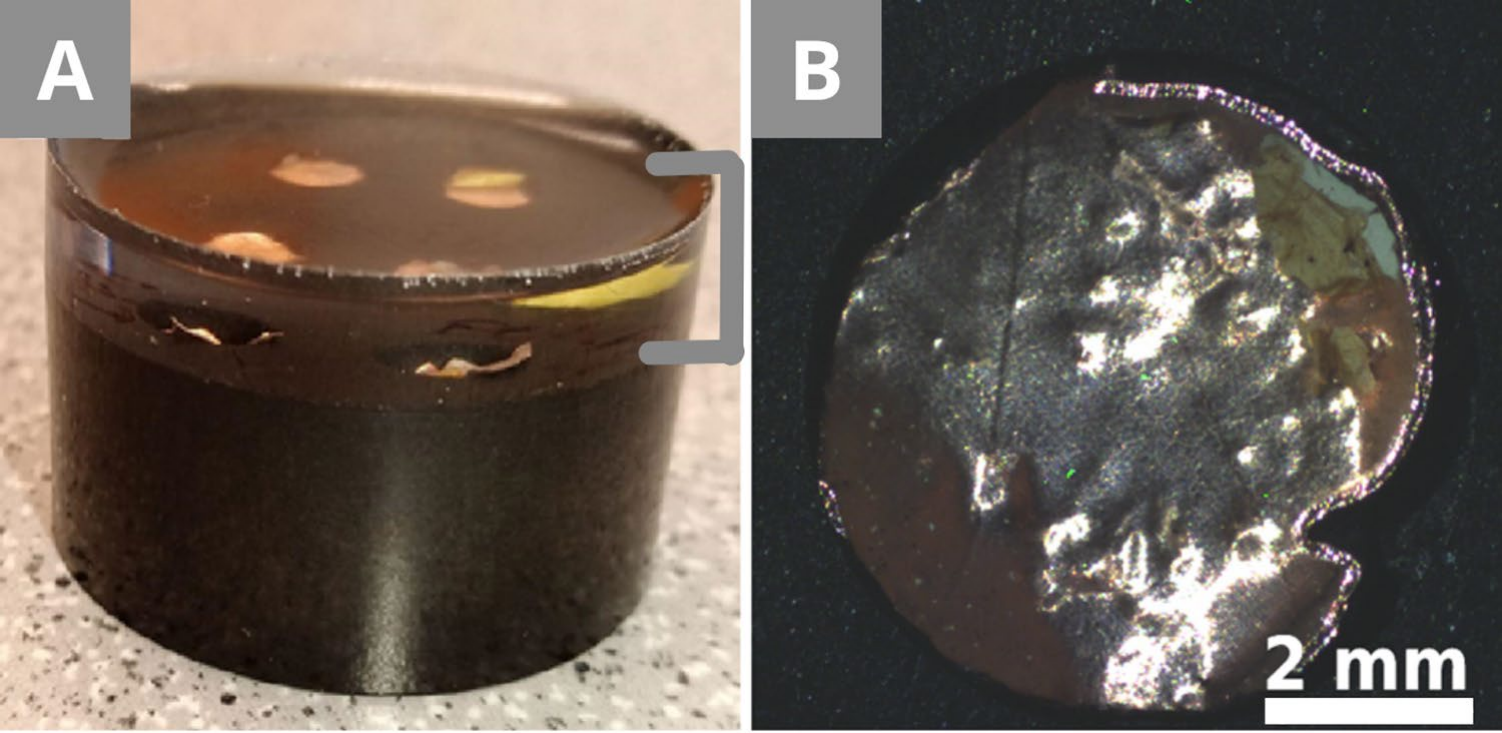
Preparation B. **(A)** Transparent resin fills the holes made in the conductive resin. This layer can be reduced to its minimum by filling only the holes. The thickness of the transparent layer of the cross-section (marked grey) and the conductive resin can be used for automatic polishing. **(B)** The sample is fixed with copper tape.

Preparation A reduces the manipulation of the sample (Fig. 7). The diameter and depth of the hole can be adapted to the size and shape of the sample. In our case, a diameter of 5 mm and a depth of 2 mm were used (Fig. 7A). However, the diameter was too large for our samples, which were between 1 and 3 mm long (Fig. 7B). The chosen depth was relatively advantageous for all samples.

Under the microscope, scratches were visible on the surface of the cross-sections A. There was no visible difference in the depth of the scratches between the reference cross-sections and cross-sections A. These scratches occur due to the combination of coarse polishing particles and the pressure applied during the polishing steps (Fig. 6A). It could be visually attenuated by applying a drop of ethanol or water and a coverslip on top (Fig. 6B), but this technique is not recommended, as it can dissolve some components of the sample.

Preparation A has a quick curing time, but includes preparatory steps that need to be planned relatively in advance (drilling, two rounds of curing). It also needs two different resins (PolyFast, EpoFix). The thickness of the transparent resin can be measured (Fig. 7A) and used to set the parameters of the automatic polishing to protect the sample from being destroyed during the process. It helps change the polishing mode from wet to dry in time. The holder used to secure the cross-section while polishing them reduces uneven surface, deep scratches, and offers the possibility to set in the automatic mode the polishing time and pressure in the Tegramin. For manual polishing, the size of the holder offers a better hold of the cross-section for the users, which improves the polishing results. The main advantage of Preparation A is the possibility of preparing more samples simultaneously. In the case of preparing multiple samples, the amount of the PolyFast resin is reduced compared to a single sample preparation. Moreover, with all samples polished into a uniform height, there is no need to adapt the focus to each cross-section separately, which reduces the working time. Hence, one cross-section containing several micro-samples can reduce the preparation time, measurement time, and amount of material (i.e. embedding medium).

Preparation B (Fig. 4) also requires the first half of the EpoFix resin to be cured in advance and its surface coated with carbon (appr. 6–8 h). Both steps can be carried out in a batch and stored for later use. This preparation does not need any additional materials or procedures. Fixing the sample is the most sensitive part of the procedure, and it strongly influences the conductivity of the preparation, as the fixing medium acts as an insulator. The second coating needs to be connected to the first coating layer, and this carbon network should be in contact with the sample all around, but also with the SEM metallic holder to create an appropriate exit path for the electrons to avoid charging. The cross-section polishing becomes quicker and easier with the holder and the Tegramin. The pressure control of the Tegramin can also help reduce the scratches.

The capsule-embedding reference preparation was easy, quick, and cheap, and had only 2 steps (sample positioning, resin curing). The main issues of this preparation were the sample drifting during curing, the long curing time because of the relatively large amount of the resin, and the charging of the cross-section under the SEM beam. With the flat-embedding preparation, which was used for Preparation B, the positioning was easier, but there were three steps (resin curing, positioning, resin curing again) and it was also charging. Preparation A was the most stable and easiest for sample positioning.

### Cross-section conductivity

**Figure 8:**
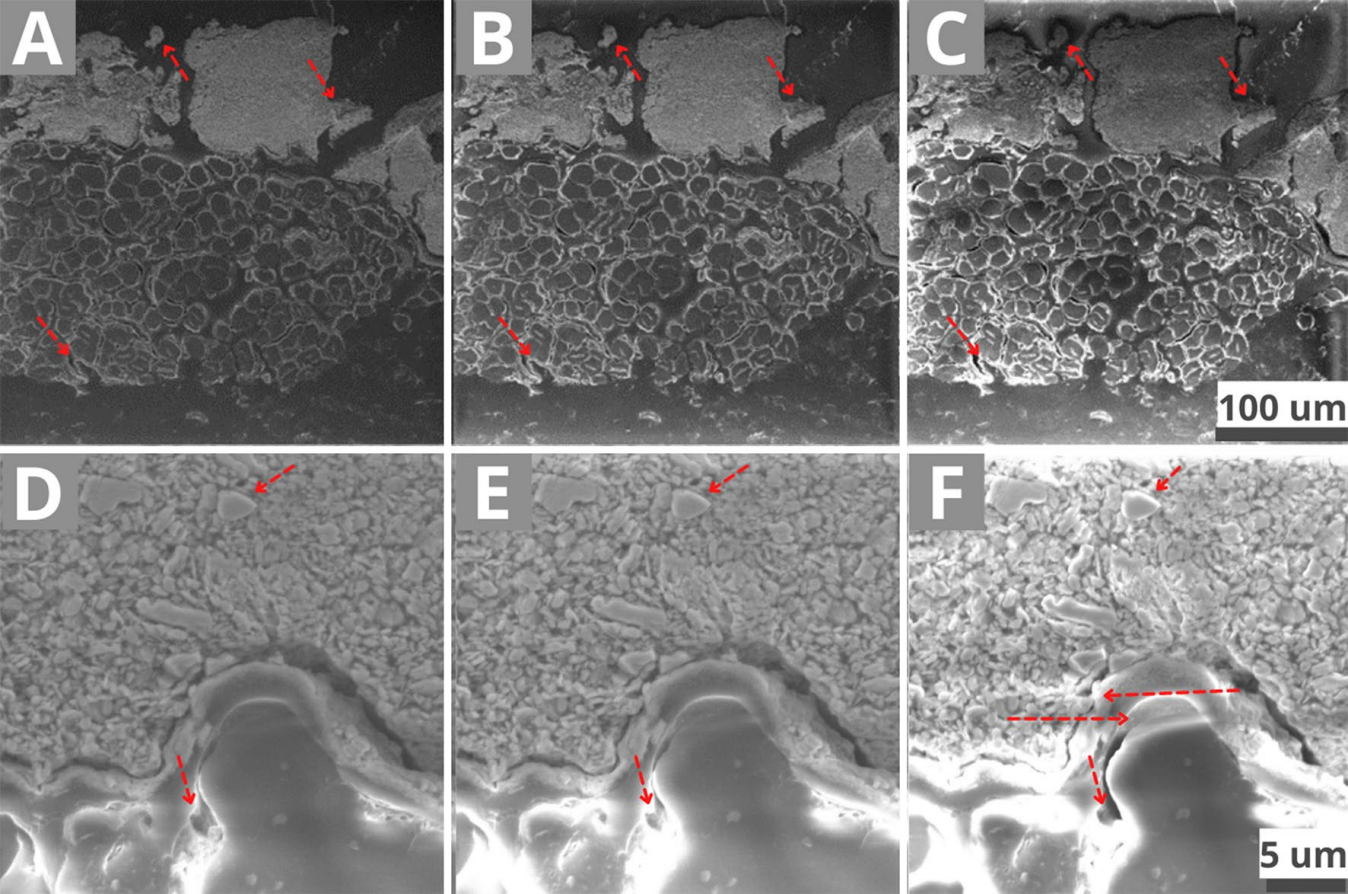
RS1 reference at 10 kV average magnification (801 x) WD 14 mm **(A)** 50 Pa vacuum marked as 1—low charging. The image contrast is relatively homogeneous, and the image is relatively stable. **(B)** 20 Pa vacuum marked as 2—Medium charging. Some parts of the pictures are becoming bright or dark (red arrows), the image contrasts are less homogeneous, and the image is unstable. **(C)** 15 Pa vacuum marked as 3—High charging. Very bright or dark parts hiding details of the sample appear, the image contrast is inhomogeneous, and the image is highly unstable (drifting). The contrast manager cannot smooth the differences. RS1 reference at higher magnification (10 kx) **(D)** 50 Pa 1—low charging. Some parts are charging (organic and resin; uneven brightness (Fig. 1A), but the image quality stays acceptable. (E) 20 Pa 2—Medium charging. Most details are invisible due to the increasing uneven brightness and the contrast of the inorganic material increases (red arrows). The image drifting increases. (F) 15 Pa 3—High charging. The uneven brightness is now on all materials. Bright lines (horizontal arrows; Fig. 1B) and a blurred image can also be observed.

The charging of the cross-sections was visually assessed and marked from 0 to 3: 0 – no charging; 1—low charging and a relatively homogeneous image contrast Fig. 8A + 8D; 2—half of the components are charging, and some charging effects (Fig. 1) appear more consistently Fig. 8B + 8E; 3—more than ¾ of the components are charging, and charging effects are strongly occurring Fig. 8C + 8F. In Fig. 8C and 8F, a high over-exposition (electron concentration) of the materials can be observed, particularly of the fibres (organic), but also a darkening of the inorganic material rims, creating a contrast imbalance. The uneven brightness (Fig. 1A) of the material creates visual artefacts and hinders proper observation of the sample. From Fig. 8A–C and at higher magnification from 8D to 8F, a contrast gradation from homogeneous to inhomogeneous can be seen.

The reference preparation showed high charging (2–3) at 10 kV, but stabilised from 20 kV (2–1). A charging modification depending on the sample morphology occurred around 30 Pa. The homogeneous samples (MS1/2) had stable charging effects on their surface compared to the multi-layered ones (MS3, RS1/2 Fig. 8A), where the charging was high in localised areas (Fig. 8D—around organic fibres). The charging was reduced from 3 to 2, but was still present at any magnification (low 500 x; average 500 × to 3 kx; high 3 kx), and with any parameters and samples (1).

**Figure 9:**
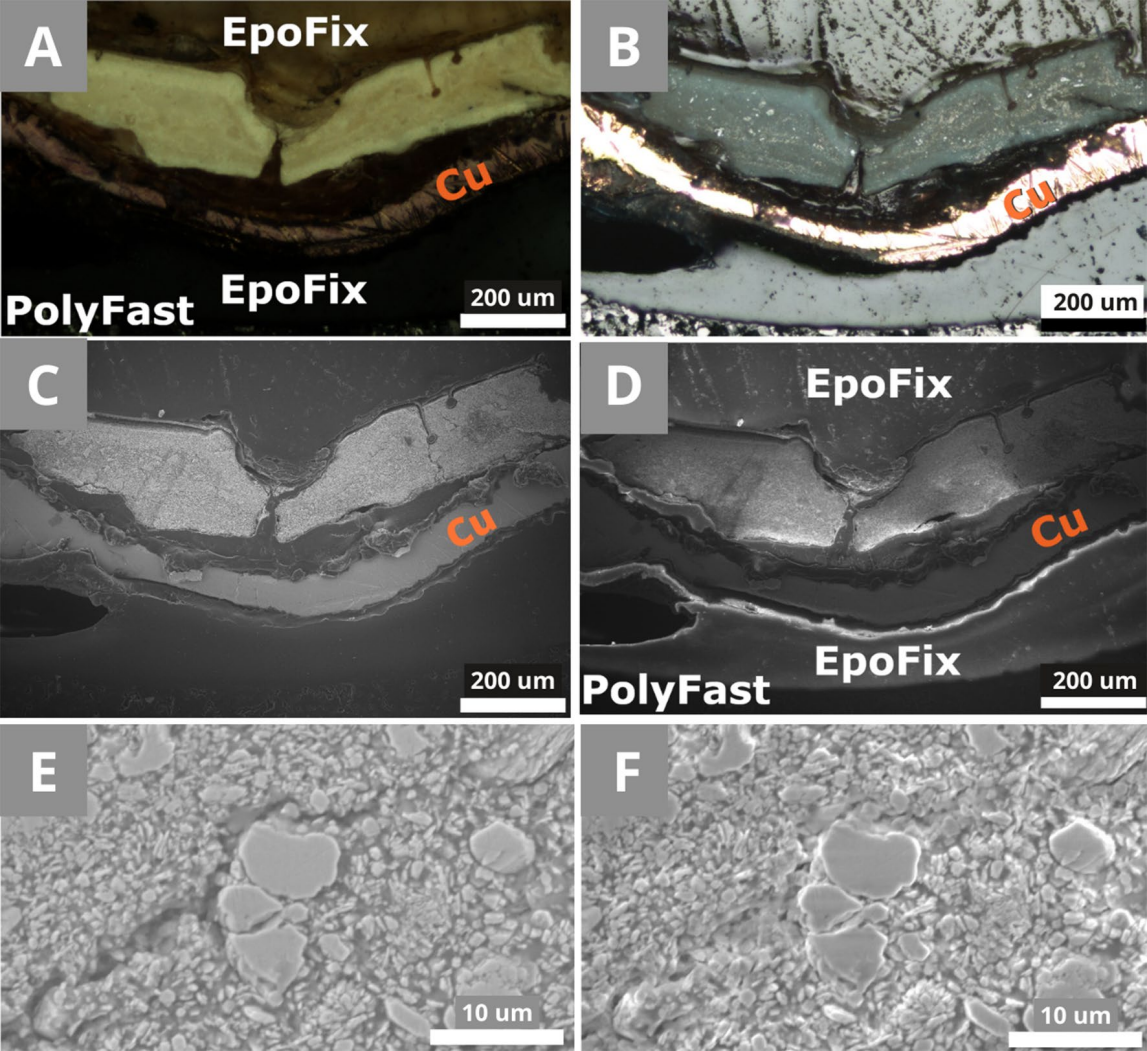
RS1 in preparation A. **(A)** Bright light and **(B)** polarized light microscopic image. **(C)** SEM-SE image at 10 kV | 50 Pa and **(D)** at 10 kV | 20 Pa. **(E)** SEM-SE image at 10 kV | 50 Pa and **(F)** at 10 kV | 20 Pa at a higher magnification.

In Preparation A (Fig. 3), the sample is in direct contact with a conductive material. The copper tape (Cu) (Fig. 9A + 9B) is connected to the conductive resin, creating an exit path for the electrons. At 10 kV | 10 Pa and 15 Pa, the charging in the middle of the non-conductive resin was very high (3) at low magnification and slightly lower (2) at an average magnification. Almost no charging occurred at 10 kV | 50 Pa, even at a high magnification (1–0) (Fig. 9C), whereas in the reference preparation 0 charging effect was never reached. Both the homogeneous and the multi-layered samples had a charging decrease, 2 to 1, at 10 kV | 20 Pa (Fig. 9D). This preparation showed promising results, where the charging occurred only in the transparent resin area, but not around the sample or the copper tape. At a high magnification, the charging was reduced only where the sample was in direct contact with the conductive material (Fig. 9D). Even though, at a high magnification, the charging of the chalk ground decreases to a certain extent due to a vacuum increase (Fig. 9E + 9F). The sample needs to be in tight contact with the conductive component to effectively reduce the charging.

**Figure 10:**
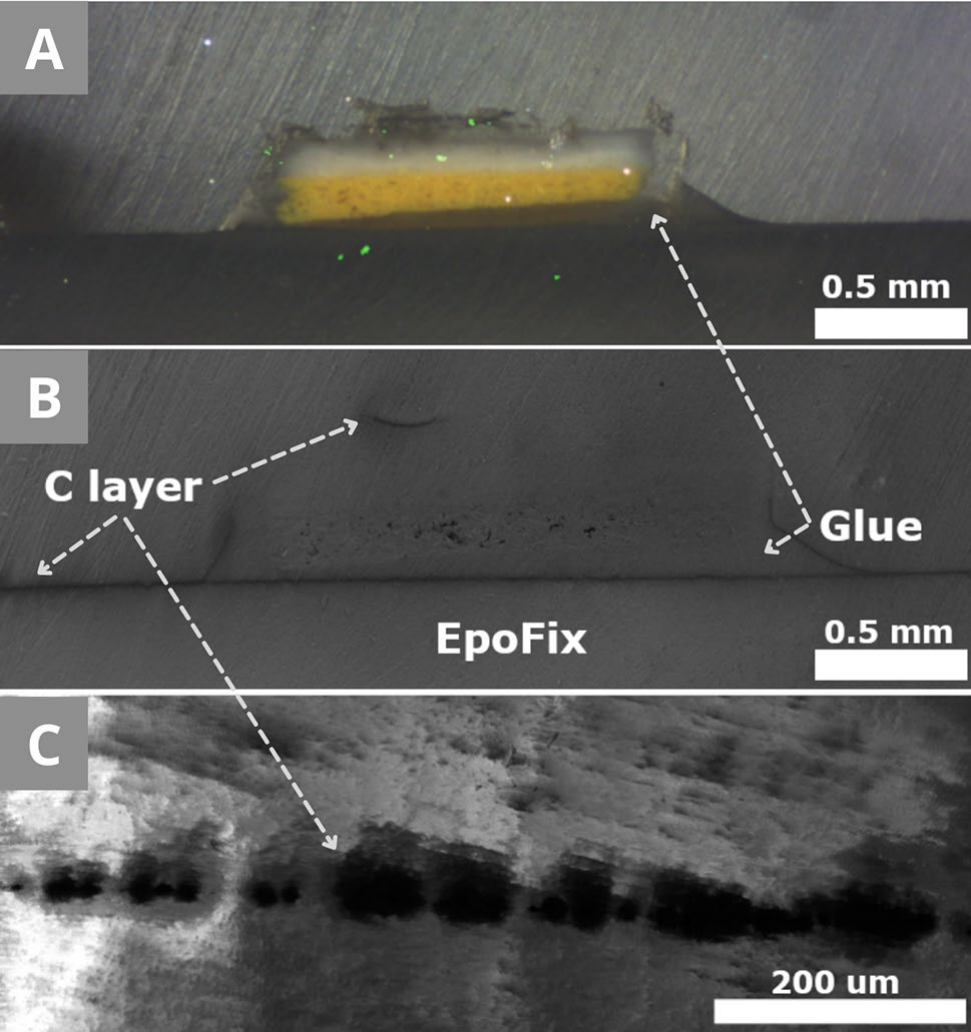
Preparation B of MS2. **(A)** Bright light microscope image. The sample is not in contact with the first C-layer. The green speckles are due to the camera. **(B + C)** SEM-SE + BSE imaging 2 kV | 10 Pa. **(B)** The carbon line is broken around the sample and it is not regular. **(C)** The detail of the uneven carbon layer of (B).

Preparation B (Fig. 4) was designed based on the observations made during Preparation A. The use of carbon sputtering for the coating fits the shape of the sample and surrounds it better than the copper tape (Fig. 10A). The direct contact between the conductive material and the sample was expected to be better than in the Preparation A. The coated surface of the resin (Fig. 4C) works as the exit path for the electrons from the cross-section to the metallic SEM holder. Similarly, in Preparation A, the charging was reduced where the sample was in direct contact with it. But the carbon layer was not even (Fig. 10B) and was disrupted during the sputtering mostly around the samples' edges and vertical surfaces. During the carbon sputtering, the vacuum can be chosen, as well as the theoretical thickness of the carbon layer. In our case, 30 nm thickness (relatively thick) was chosen for a low vacuum (around 2 Pa). With a lower vacuum, the sputtering was less precise and less even (Fig. 10C). The main problem of this preparation was the glue spreading between the sample and the carbon layer. Therefore, the sample was mostly not in contact with the first carbon layer anymore. The second coating disruption occurred due to the direction of the sputtering (the stage of the coating device can be tilted to improve the carbon deposition depending on the sample shape). Only one side of the sample was coated, and due to its uneven surface and morphology, the connection between the two carbon layers was not as good as expected. Consequently, the charging was not appropriately reduced.

The preparations of the cross-sections A and B have several advantages over the reference cross-sections, but there is still room for improvement in both techniques concerning the charging decrease. Regarding cross-sections A, the positioning of the sample is easier with the copper tape and only a small area of a liquid resin. The extent of the sample manipulation is greatly reduced due to the holes drilled in the conductive resin. The low amount of the resin reduces the formation of bubbles, and the copper tape at the bottom of the sample does not interfere with its analysis. The curing time is also quicker in Preparation A, because of the small amount of the transparent resin needed. The copper tape does not fit the morphology of all samples well, increasing the risk of breaking the sample, to fit it better for enough contact to avoid charging effects. In our case, the holes were drilled, but they could have been melted with a soldering iron tip, which might be easier to use in a laboratory and enable to shape the hole with regards to the sample morphology. This would reduce the use of the resin, the size of the hole, and therefore the charging, as the conductive layer would be much closer to the sample.

Regarding cross-sections B, the positioning and curing time are similar to the reference flat-embedding. In both cases, if the first parts (curing of the first part of the resin, carbon coating of its surface) of the cross-sections are prepared in advance as a batch, the time of the preparation of the cross-section is cut in half. Glue was used to fix the samples on the first cured part of the resin, but it limited the contact with the coated surface and partially neutralised the effect of the charging reduction. Based on our findings, the use of carbon tape or carbon paint instead of normal glue to fix the sample in step D in Fig. 4 is probably a better choice and needs to be tested. This helps positioning the sample and decreasing the charging, as well as connecting the conductive parts around it. The cross-section B showed a slight reduction of charging where the sample was in contact with the carbon coating, but the connection between the two layers of carbon was not consistent around the sample, as already stated. This preparation needs further improvements regarding the carbon coating connection, but based on our observations, it should show better results in decreasing the charging and easing the positioning of the sample in fewer preparation steps. Cross-section A is currently the most advanced and gives the best results from the three preparation methods concerning the charging reduction, the sample positioning, and the preparation time. For all cross-sections, the best vacuum and voltage combination for the lowest charging effect was 50 Pa and 10 kV, respectively, regardless of the sample. The reference preparation is common, but achieving a 0-charging effect with such preparation can be tricky without risking the sample integrity. The cross-sections A and B showed a charging decrease, which could lead to a 0-charging effect with the small improvements proposed in this work, without impact on the sample surface to be analysed.

## Conclusion

Different methods of cross-section preparation of valuable painting micro-samples were explored in this work. Such cross-sections should not be covered with a coating that hinders light-microscopic observation and can hardly be removed. The addition of carbon paint on top of the cross-section works well around the area where the paint touches the sample, or when the sample includes a conductive paint layer. This work aimed to improve the preparation of non-conductive samples and to decrease the charging with common laboratory materials and devices, which offer easy reproducibility of these preparations. Preparation A (conductive resin + copper tape + transparent resin) enabled a significant charging decrease, but could not fully remove it. Preparation B (transparent resin + 2 side coatings) has a bigger potential to remove charging, but needs further testing and improvements, such as a better connection of the carbon layers. The preparation process can be further improved, but this study proved that the preparation of a conductive cross-section for non-conductive sensitive samples works without altering their surface characteristics, morphology or material composition for LM and SEM analyses.

## Data Availability

The datasets generated and analysed during the current study are mainly included in this article, and complementary data are available from the corresponding author on reasonable request.
