# Publisher Correction: Conductive cross-section preparation of non-conductive painting micro-samples for SEM analysis

**DOI:** 10.1038/s41598-022-26752-4

**Published:** 2022-12-28

**Authors:** Victory Armida Janine Jaques, Eva Zikmundová, Jiří Holas, Tomáš Zikmund, Jozef Kaiser, Katarína Holcová

**Affiliations:** 1 Institute of Geology and Palaeontology, Faculty of Science, Charles University, Albertov 6, 12843 Praha 2, Czech Republic; 2 CEITEC - Central European Institute of Technology, Brno University of Technology, Purkyňova 656/123, 612 00 Brno, Czech Republic

Correction to: *Scientific Reports* 10.1038/s41598-022-21882-1, published online 16 November 2022.

The original version of this Article contained errors in the Affiliations.

Eva Zikmundová, Jiří Holas, Tomáš Zikmund and Jozef Kaiser were incorrectly affiliated with ‘Institute of Geology and Palaeontology, Faculty of Science, Charles University, Albertov 6, 12843 Praha 2, Czech Republic’.

The correct affiliation for Eva Zikmundová, Jiří Holas, Tomáš Zikmund and Jozef Kaiser is listed below.

CEITEC - Central European Institute of Technology, Brno University of Technology, Purkyňova 656/123, 612 00 Brno, Czech Republic.

Additionally, Katarína Holcová was incorrectly affiliated with ‘CEITEC - Central European Institute of Technology, Brno University of Technology, Purkyňova 656/123, 612 00 Brno, Czech Republic’.

The correct affiliation for Katarína Holcová is listed below.

Institute of Geology and Palaeontology, Faculty of Science, Charles University, Albertov 6, 12843 Praha 2, Czech Republic.

The original Article has been corrected.

